# Phase II Multicenter Clinical Trial of Intraoperative Molecular Imaging with Abenacianine During Lung Cancer Surgery

**DOI:** 10.1245/s10434-026-19951-0

**Published:** 2026-07-26

**Authors:** Luis J. Herrera, Gavin M. Wright, Jae Y. Kim, Janani S. Reisenauer, David C. Rice, Sunil Singhal

**Affiliations:** 1 Orlando Health Cancer Institute, Orlando, FL; 2 St. Vincent’s Hospital, Melbourne, Australia; 3 City of Hope National Medical Center, Duarte, CA; 4 Mayo Clinic, Rochester, MN; 5 University of Texas MD Anderson Cancer Center, Houston, TX; 6 Department of Surgery, University of Pennsylvania Perelman School of Medicine, Philadelphia, PA

**Keywords:** Non-small-cell lung cancer, Near-infrared imaging, Surgical imaging, Intraoperative molecular imaging, Fluorescence-guided surgery, Surgical margins

## Abstract

**Background:**

Surgery is the cornerstone for locally contained lung cancer; however, intraoperative challenges include accurate lesion localization and assessment of surgical margins. Intraoperative molecular imaging (IMI) using near-infrared fluorescence-guided surgery is a real-time tumor-targeted optical imaging technique. A multicenter phase II clinical trial evaluated abenacianine (VGT-309), a cathepsin-targeted near-infrared (NIR) IMI agent for visualization of pulmonary nodules and margins during surgery. The primary objective was to assess the efficacy of abenacianine.

**Methods:**

At six sites, patients scheduled to undergo surgery for known or suspected cancer in the lung received intravenous abenacianine 0.32 mg/kg 12–36 h before surgery. Efficacy was measured by the proportion of clinically significant events, defined as localization of lesions not found by standard surgical techniques, identification of additional cancers, identification of inadequate surgical margins confirmed by histology, and detection of cancerous lymph nodes.

**Results:**

Of 89 patients who received abenacianine and surgical resection, 40 (45%) had at least one clinically significant event. Abenacianine with NIR imaging identified lesions not found by standard methods in 33 (37%) patients, synchronous and occult cancers not found by preoperative imaging in three (3%) patients, margins within 10 mm of the closest staple line in eight (9%) patients, and cancerous lymph nodes in one (1%) patient. Abenacianine was safe and well-tolerated in this study; there were no drug-related serious adverse events.

**Conclusions:**

IMI with abenacianine during standard-of-care lung cancer surgery improved intraoperative localization, identification of occult lesions, margin assessment, and lymph node detection, enabling a more complete oncologic resection.

Lung cancer is a particularly lethal cancer for which the best chance of cure is early detection and complete surgical resection. Surgery is a cornerstone of curative treatment for most solid tumors, and successful surgery relies on the ability to differentiate cancer tissue from healthy adjacent tissue; however, overall positive margin (residual retained cancer cells) rates across cancer types have been observed to range from 15% to 60%.^1^ After lung cancer resection, positive margins generally occur in 5–15% of non-small-cell lung cancers (NSCLCs).^2,3^ Sublobar resections are more likely to have positive margins than lobectomies.^4,5^ In NSCLC, residual retained cancer cells correlate with negative outcomes, including poor survival.^6^

Minimally invasive surgery (MIS), such as video-assisted or robotic-assisted thoracic surgery, is being used with greater frequency for resection surgeries, including cancer in the lung, because it confers benefits such as decreased tissue trauma, pain, blood loss, length of hospital stays, and postoperative complications. Unlike open surgical approaches, MIS limits tactile feedback for the surgeon, making it difficult to localize nodules, confirm adequate margins, and identify synchronous lesions.^7^

Molecular targeted agents have revolutionized many areas of cancer diagnosis and treatment, including surgery. For example, intraoperative molecular imaging (IMI) is a novel technique that entails performing real-time, in vivo optical imaging by injecting a tumor-targeted optical contrast agent that allows the surgeon to visualize cancer cells during surgery to ensure disease clearance. Specifically, IMI uses a tumor-targeted, fluorescent imaging agent that can be taken up and bound by cancer tissues and seen by tumor-specific fluorescence under near-infrared (NIR) light during the surgical procedure, which can render the lesion readily identifiable for the surgeon. This allows early and well-targeted surgical biopsy and subsequent appropriate cancer surgery for the correctly identified population.

Existing IMI agents approved by the US Food and Drug Administration are either not targeted (e.g. 5-ALA), have limited efficacy for diverse cancer subtypes, or are not compatible with most clinical camera systems in the USA (e.g. pafolacianine). Abenacianine (VGT-309) is a tumor-targeted, activatable, fluorescent intraoperative imaging agent that can be visualized with all existing indocyanine green (ICG)-compatible NIR imaging systems. The molecule covalently and irreversibly binds to active cysteine cathepsins, a family of proteases enriched in a broad range of solid tumors, including NSCLC.

Following successful safety studies in healthy human volunteers,^8^ a dose-ranging clinical trial in patients with cancer in the lung,^9^ and a single-center phase II clinical trial evaluating the safety and efficacy of IMI with abenacianine for the identification of cancers in patients undergoing pulmonary resection,^10^ a multi-center phase IIb clinical trial was conducted using abenacianine for injection to visualize pulmonary lesions during surgery. The primary objective was to assess the efficacy of abenacianine in localizing pulmonary lesions, evaluating surgical margins, and identifying unsuspected disease.

## Materials and Methods

### Patient Selection

This phase II, multi-center, open-label clinical trial (NCT06145048; https://clinicaltrials.gov/study/NCT06145048, version 1 submitted 17 Nov 2023) was approved by the University of Pennsylvania, MD Anderson Cancer Center, Orlando Health Cancer Institute, Mayo Clinic, and City of Hope institutional review boards and St. Vincent’s Hospital (Melbourne) human research ethics committee. Patients scheduled to undergo surgical resection of a proven or suspected cancerous lung nodule or mass for diagnostic or curative intent were counseled by the surgeon for study inclusion. Patients enrolled in the study met all preoperative surgical and anesthesia acceptance criteria, were aged ≥18 years, were willing and able to sign the informed consent and comply with study procedures, were male or female of non-childbearing potential or willing to use highly effective contraception, had acceptable kidney and liver functions per protocol definition, and had an Eastern Cooperative Oncology Group score of 0–2. All patients gave informed consent for inclusion in the study and publication.

### Study Design

Patients were administered abenacianine 0.32 mg/kg between 12 and 36 h before surgery by intravenous infusion over 15–20 minutes using a syringe pump. Patients were observed for 1 h after dosing and asked about possible treatment-emergent adverse events.

During each surgery, the surgeon utilized standard surgical techniques of white light inspection and/or palpation followed by in vivo NIR fluorescence imaging using an NIR imaging system capable of detecting ICG for localization of the primary tumor and any potential synchronous or occult lesions and lymph nodes.

The numbers and locations of pulmonary nodules and synchronous lesions that were visualized by standard techniques and/or by abenacianine with NIR fluorescence imaging were recorded. Lymph nodes visualized by abenacianine NIR fluorescence imaging were identified and recorded by nodal station.

Following extraction of any tumors/nodules, ex vivo NIR fluorescence imaging to measure the surgical margin was performed in the operative suite before the surgical field was closed.

The primary efficacy endpoint was to determine the proportion of patients that had at least one clinically significant event (CSE), defined as follows:

1. Intraoperative localization of one or more preoperatively identified lung lesions using abenacianine with NIR imaging when standard surgical techniques using white light and/or palpation failed to identify the lesion(s), and lesion(s) are confirmed by histologic examination to be other than normal lung tissue.
2. Identification of a histologically confirmed cancerous or precancerous synchronous/occult lesion using abenacianine with NIR imaging when standard surgical techniques using white light and/or palpation and preoperative imaging failed to identify the lesion(s).
3. Identification of fluorescence within ≤10 mm from the inside edge of the closest staple line as measured by the investigator ex vivo in the operating room using NIR imaging, with pathologic margin confirmed by histologic examination to be ≤10 mm. Margins from excisions of known or suspected metastases at the time of the surgery, or diagnostic wedge resections where a completion lobectomy or segmentectomy was subsequently performed, were not considered for margin CSE inclusion.
4. Identification of lymph nodes by abenacianine with NIR imaging confirmed by histologic examination to be cancerous.

The secondary efficacy endpoints evaluated the sensitivity, positive predictive value (PPV), and false discovery rate (FDR) of abenacianine with NIR imaging to identify malignant (cancerous or precancerous) lesions and lymph nodes in vivo as defined below:

1. Sensitivity is defined as the probability that the tissue fluoresces when it is cancer, as confirmed by history ((TP/(TP+FN)), where TP means “true positive” and FN means “false negative.”
2. PPV is defined as the probability that a tissue sample contains cancer on histologic exam if it fluoresces ((TP/(TP+FP)), where FP means “false positive.”
3. FDR is defined as the probability that a tissue sample does not contain cancer when it fluoresces ((FP/(TP+FP)).

The exploratory endpoints evaluated the sensitivity, PPV, and FDR of abenacianine with NIR imaging to identify malignant lesions ex vivo.

The false positive rate (FPR, FP/(FP+TN), where TN means “true negative”) for lymph node fluorescence by abenacianine with NIR imaging, the probability that a lymph node fluoresces when it does not contain cancer, was calculated post hoc.

### Molecular Imaging Agent

Abenacianine (chemical formula C_127_H_142_ClF_4_N_10_Na_3_O_23_S_5_; molecular weight, 2517.29 Da) is a quenched, activity-based molecular imaging agent that consists of a phenoxymethyl ketone electrophile that covalently and irreversibly binds active cysteine cathepsins, coupled to an ICG fluorophore and an IRDye QC-1 quencher (LI-COR Biosciences, Lincoln, NE, USA). The quencher is released upon binding the cathepsin to allow fluorescence from the agent. The abenacianine drug substance was manufactured in compliance with good manufacturing practice by Chinese Peptide Company (Hangzhou, Zhejiang, China). The abenacianine drug product was manufactured in compliance with good manufacturing practice by UI Pharmaceuticals (Iowa City, IA, USA).

### Near-Infrared (NIR) Fluorescence Imaging Systems

Abenacianine can be used for imaging with a broad range of ICG-enabled devices. Real-time fluorescent imaging was performed in vivo using at least one of the following imaging systems: da Vinci Xi Surgical System with Firefly Fluorescence Imaging and X/Xi Endoscope Plus (Intuitive, Sunnyvale, CA, USA), da Vinci SP Surgical System with Firefly Fluorescence Imaging (Intuitive), EleVision IR Platform (Medtronic, Minneapolis, MN, USA), Stryker 1788 AIM 4K Platform (Stryker Endoscopy, San Jose, CA, USA), Stryker 1688 AIM 4K Platform (Stryker Endoscopy), IMAGE1 S Rubina for NIR/ICG imaging (Karl Storz SE & Co. KG, Tuttlingen, Germany).

### Statistical Analysis

Efficacy analysis included all 89 patients administered abenacianine who underwent standard-of-care lung surgery using white light and/or palpation followed by IMI (intent-to-treat population). For the primary efficacy endpoint of proportion of subjects with at least one CSE, assuming a 20% true rate of at least one CSE, two-sided 5% type 1 error rate and 80% power, the sample size to rule out a CSE rate ≤10% requires 86 evaluable subjects in the primary analysis group. The trial will have met the primary endpoint if at least 15/86 (17.4%) subjects have at least one CSE. The 95% two-sided Clopper–Pearson exact confidence bound at 17.4% is (10.1%, 27.1%). This single arm trial is powered at 80% to rule out ≤10% of subjects experiencing at least one CSE.

A descriptive analysis of the baseline subject and tumor characteristics was performed. Quantitative data were presented as a standard set of summary statistics (n/mean [standard deviation]/median/minimum/maximum). Categorical data were expressed as frequency (%). For the primary endpoint analysis, the proportion of subjects undergoing NIR imaging with at least one CSE was identified and tested under the following null hypothesis: Ho: P<0.10. The alternative hypothesis states that the proportion of subjects undergoing NIR imaging with at least one CSE was greater than the chosen margin of 0.10: Ha: P>0.10. At 86 evaluable subjects, this trial is powered at 80% to reject the null hypothesis of ≤10% subjects experiencing at least one CSE. Two-sided 95% exact confidence intervals for the proportion of subjects with at least one CSE was constructed.

For the secondary endpoint analysis sensitivity, PPV, and 1-PPV was calculated and presented along with 95% confidence intervals using generalized estimating equation model analysis to factor in within-subject correlation for subjects with multiple tumors.

The safety analysis included all 89 patients who received abenacianine (safety population) and was assessed using standard guidelines (https://ctep.cancer.gov/protocolDevelopment/electronic_applications/ctc.htm).

## Results

### Patient Characteristics

**Fig. 1:**
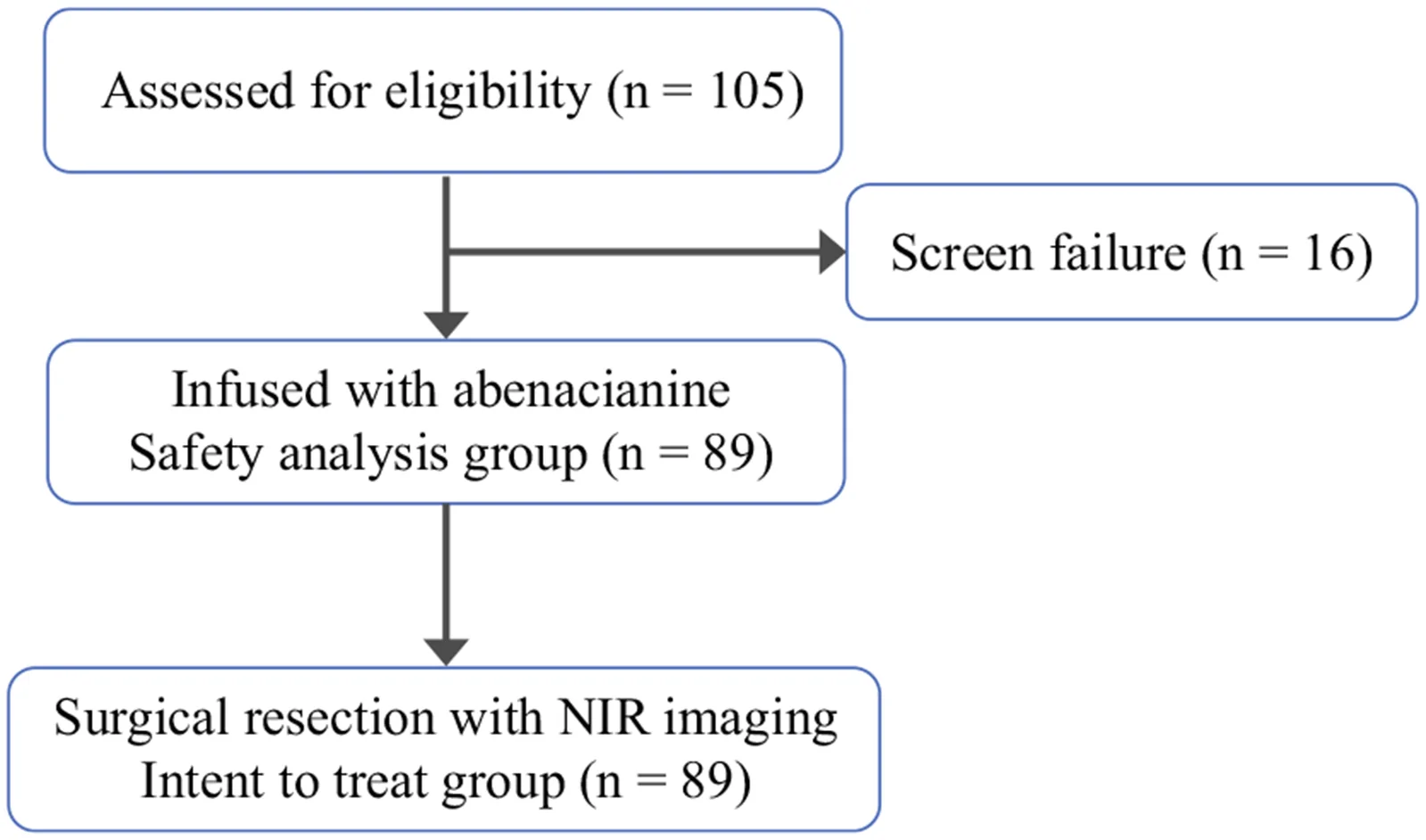
Consolidated Standards of Reporting Trials (CONSORT) diagram. NIR, near-infrared

A total of 89 of the 105 screened patients with confirmed or suspected cancer in the lung met eligibility requirements and were enrolled in the study. All 89 enrolled patients were administered abenacianine and underwent surgical resection using NIR fluorescence imaging and were included in the safety and intent-to-treat populations. The Consolidated Standards of Reporting Trials (CONSORT) flow diagram is shown in Fig. 1.

Demographic characteristics of the safety population (*N* = 89) are presented in Table 1. The mean age of patients was 66.9 years and ranged from 18 to 85 years; 65 patients (73%) were aged ≥65 years. A majority were female (57 [64%]) and white (70 [78.7%]). Eleven (12.4%) patients were current smokers, 45 (50.6%) were former smokers, and 33 (37.1%) had never smoked. Of the 56 current and previous smokers, mean years of smoking was 32.3. The study population included patients with both primary and secondary lung cancer. Of the 64 patients with primary lung cancer, 58 had stage I (90.6%), three had stage II (4.7%), two had stage III (3.1%), and one had stage IV (1.6%). Twenty patients had secondary lung cancer, including metastatic breast, colorectal, prostate, thymoma, renal cell, and sarcoma, and one patient had lymphoma. Four patients did not have malignant lesions. Of the 141 resected lesions that were imaged in vivo, 107 were cancerous and 34 were noncancerous. Malignant tumor size measured by pathology averaged 14.3 mm (range 3–62). All procedures were minimally invasive, and sublobar resections were performed in 74 of the 89 cases (83.1%). The remaining 15 pulmonary resections were either a lobectomy (nine), wedge with completion lobectomy (five), or segmentectomy with completion lobectomy (one). A list of surgical procedures is provided in Table 2.

### Safety Outcomes

All patients were assessed for potential treatment-emergent adverse events (TEAEs) after the start of abenacianine dosing through the end of study visit (25–35 days after administration). Of a total 385 reported TEAEs, 13 (3.4%) were considered related to abenacianine. Of the 89 subjects treated, six (6.7%) had 13 study drug-related TEAEs. The most common study-drug-related TEAEs were postoperative increases in alanine tranaminase, amylase, aspartate aminotransferase, and gamma-glutamyl transferase, which occurred in four subjects. Increases were classified as grade 1, 2, or 3, and all but one resolved to normal values by the end of the study. The gamma-glutamyl transferase value in one subject resolved to grade 1 at the 2-week post-dose visit, but they missed their final blood draw at the end of the study. Changes in liver function were attributed to study drug out of an abundance of caution. However, each of these subjects underwent intubation and a significant surgical intervention under general anesthesia, and the overall rate of observed liver enzyme elevations in the postoperative period in this trial is consistent with the literature for a similar historical cohort of subjects undergoing thoracotomy,^11^ giving confidence to the overall assessment that the use of abenacianine is not independently associated with liver dysfunction. Overall, abenacianine was safe and well tolerated in this study.

### Primary Efficacy Endpoint: Clinically Significant Events

Of 89 patients who received abenacianine and underwent standard-of-care surgical resection for known or suspected cancer in the lung, 40 (44.9%; 95% CI 34.4–55.9) had at least one CSE (Table 3). The lower bound of 34.4% rules out the null hypothesis of ≤10% CSEs, *P* < 0.0001, meeting the primary endpoint. CSEs were distributed among the 40 patients as follows: 33 patients had a CSE for localization of a preoperatively identified lesion when standard techniques failed, three patients had a CSE for a synchronous lesion not found by standard surgical techniques, eight patients had a CSE for margin within 10 mm of the closest staple line, and one patient had cancerous lymph nodes identified.

**Fig. 2:**
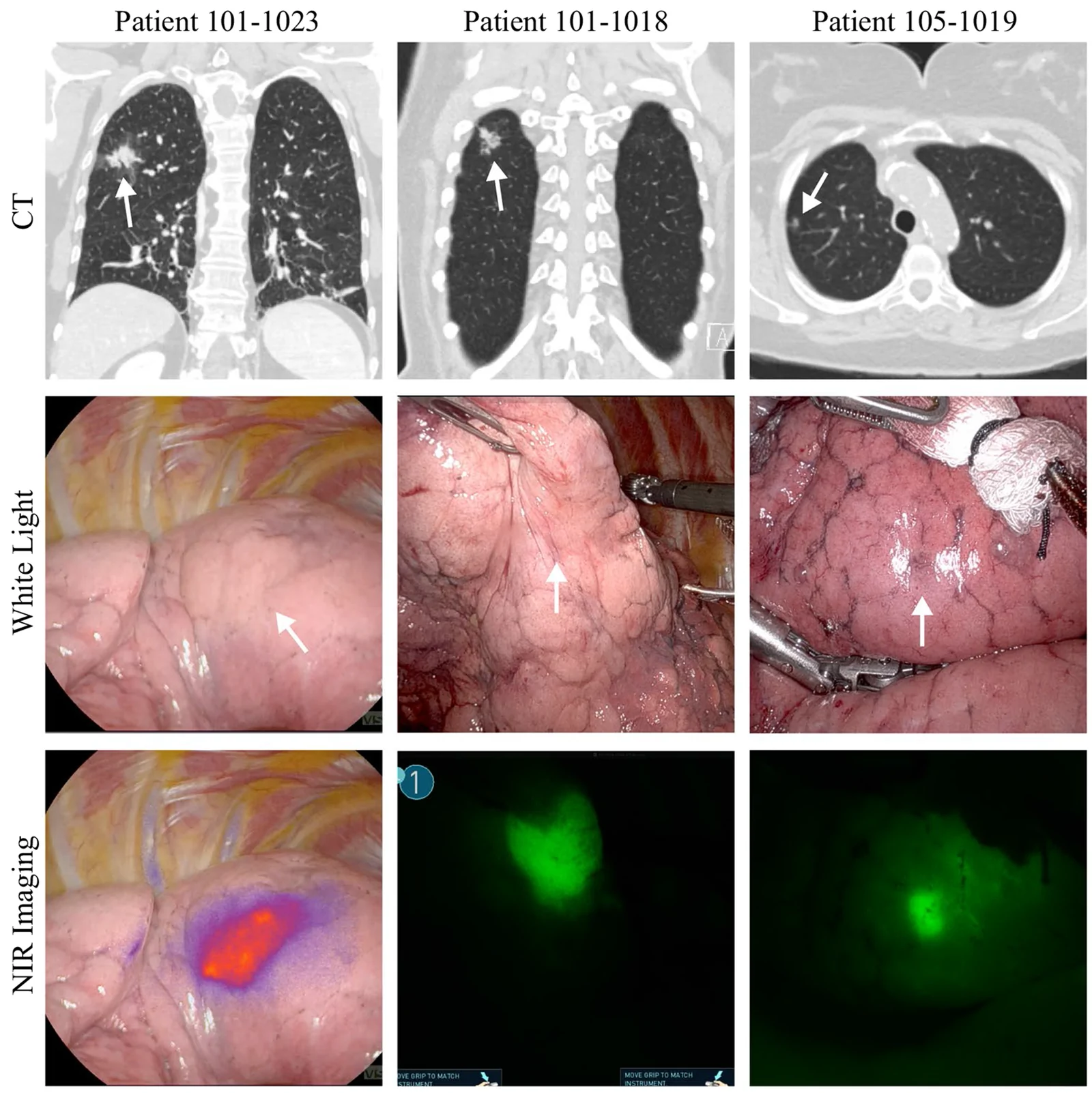
Intraoperative molecular imaging with abenacianine identifies lesions not found by standard surgical techniques. Three location clinically significant event cases are presented (left, middle, and right column). Preoperative computed tomography (CT) with pulmonary lesions of interest are indicated with white arrows (top row). In each case, white light did not identify the lesion of interest (middle row, lesions indicated with white arrows). Intraoperative near-infrared (NIR) imaging (bottom row) with the EleVision IR Platform (left column) and the da Vinci Xi Surgical System with Firefly Fluorescence Imaging (middle and left columns) identified each lesion

Of 114 lung lesions identified by preoperative imaging, 65 were successfully found using standard surgical techniques. Of 49 preoperatively identified lesions not found by white light or palpation, 38 were located with NIR imaging, one of which was excluded as a location CSE because the area that fluoresced was normal lung, resulting in 37 location CSEs (Fig. 2). Eleven lesions were not found by either method. Histopathology confirmed 36 of the 37 location CSE lesions as cancer and one as a benign hyalinized calcified nodule.

**Fig. 3:**
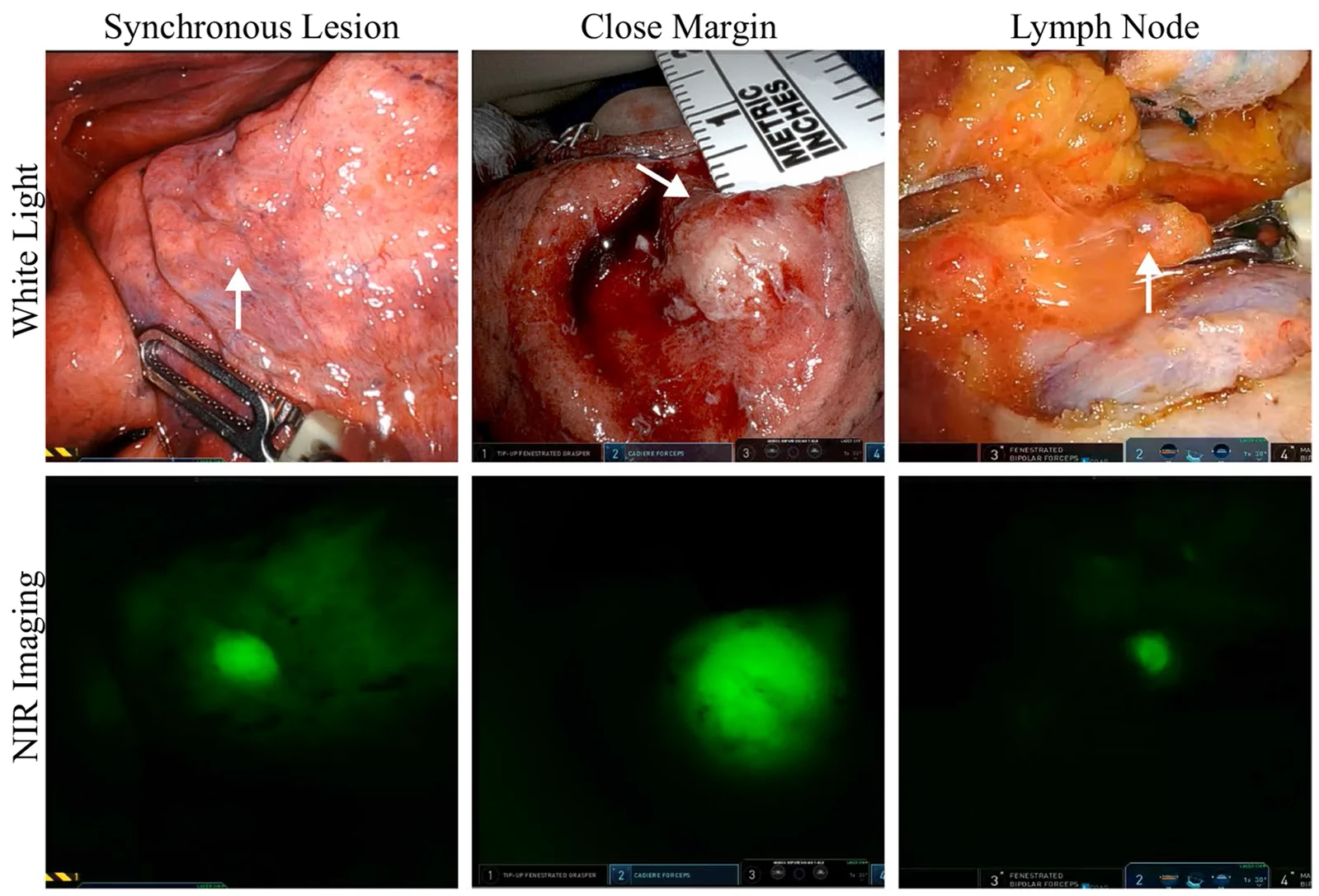
Intraoperative molecular imaging with abenacianine identifies synchronous lesions, close margins, and cancer-containing lymph nodes. Representative examples of a synchronous lesion (left column), close margin (middle column), and cancer-containing lymph node (right column) clinically significant event are presented. White light intraoperative images are shown in the top row with lesions identified with white arrows. Intraoperative near-infrared images with the da Vinci Xi Surgical System with Firefly Fluorescence Imaging are shown in the bottom row

IMI of lung and surrounding tissue identified 22 suspicious regions not found on preoperative scans or by white light visual inspection that were removed for further investigation. Three tissue samples contained malignant tumors (two lung adenocarcinomas and one liposarcoma) and qualified as synchronous lesion CSEs (Fig. 3). The remaining 19 lesions were benign. Three additional lesions not found on preoperative scans were identified by both white light and NIR imaging. Histopathology determined two of the lesions to be cancerous (one colorectal metastasis and one typical carcinoid) and one to be benign.

NIR imaging of resected tissue in the operating room allows for immediate measurement of margin distance. Of the 63 malignant lesions with margin distance measured by both NIR on the back table in the operating room and by histopathology, 27 lesions were ≤10 mm from the closest staple line when measured by NIR imaging in the operating room (Fig. 3). Of theselesions, 22 were confirmed to be ≤10 mm by histopathology measurement. Of these lesions, 10 qualified as margin CSEs.

Lymph nodes were imaged in vivo prior to removal. Only two of the 330 lymph nodes imaged contained cancer, both of which fluoresced in vivo and were from the same subject (Fig. 3).

Malignancies visualized by IMI with abenacianine included NSCLCs (adenocarcinoma, squamous cell carcinoma, neuroendocrine tumor) and cancers that metastasized to the lung (breast, colorectal, prostate, thymoma, renal cell, sarcoma).

### Secondary and Exploratory Efficacy Endpoints

The diagnostic performance of IMI with abenacianine for in vivo and ex vivo detection of cancerous lesions with NIR imaging is summarized in Table 4. To correct for multiple lesions per subject, CIs were generated using a generalized estimating equation.

A total of 141 lesions from 89 subjects were evaluated using NIR imaging in vivo. Of the 107 (TP + FN) cancerous lesions resected, 90 (TP) fluoresced in vivo, for an estimated sensitivity of 0.841 (95% CI 0.757–0.900). Of the 120 (TP + FP) lesions that fluoresced in vivo, 90 (TP) contained cancer, for an estimated PPV of 0.750 (95% CI 0.670–0.816), and 30 (FP) lesions were benign, for a FDR of 0.250 (95% CI 0.184–0.330).

The diagnostic performance of IMI with abenacianine for ex vivo detection of cancerous lesions with NIR imaging was slightly better than for in vivo (sensitivity 0.915, PPV 0.836, FDR 0.164). Of the 17 in vivo FN lesions, 11 fluoresced ex vivo. Of these lesions, 10 fluoresced ex vivo only after the tissue was bisected, suggesting that these lesions were too far from the lung surface for the fluorescence to be visualized or that the tissue content was such that the fluorescence signal was masked.

A total of 330 lymph nodes from 71 subjects were evaluated using NIR imaging in vivo. Both of the two cancer-containing lymph nodes resected fluoresced. Since only two cancer-containing lymph nodes were present in the dataset, diagnostic performance values such as sensitivity, PPV, and FDR are uninformative. However, the large number of TN lymph nodes in the study allowed us to accurately calculate the FPR – the probability that a lymph node fluoresces when it does not contain cancer. Of 328 benign lymph nodes imaged in vivo and resected, 34 fluoresced in vivo for a post hoc calculated FPR of 0.104. These data suggest that abenacianine does not nonspecifically accumulate in lymph nodes and may be a useful tool for identifying cancer-containing nodes.

## Discussion

This phase II multicenter study demonstrated that using IMI with abenacianine during standard-of-care lung cancer surgery markedly improved surgical outcomes. Abenacianine localized tumors intraoperatively, identified synchronous and occult lesions, helped assess margin adequacy, and identified cancerous lymph nodes, enabling a more complete oncologic resection.

In recent decades, the number of minimally invasive thoracoscopic and robotic-assisted lung cancer surgeries performed in the USA increased significantly, and the number of open surgeries declined.^12^ MIS procedures bring numerous clinical benefits; however, the restricted view and limited tactile clues mean that surgeons have encountered greater difficulty in localizing lesions, which increases the possibility that a tumor will be missed. Additionally, to maximize patients’ remaining lung function and reduce complications, surgeons are increasingly performing tissue-sparing (i.e. sublobar) lung resections. For surgeons to be successful in performing these procedures, the precise location of the tumor and an accurate assessment of the tumor margin must be known. Improvements in intraoperative imaging and the use of new molecular-targeted imaging agents make such accurate assessment possible.

NIR fluorescing agents show promise in enhancing the surgeon’s ability to accurately identify tumors.^13^ Novel intraoperative imaging agents, such as the recently approved pafolacianine (Cytalux), demonstrate improved tumor identification compared with conventional methods; however, limitations persist, including compatibility issues with most commercially readily available NIR imaging systems and challenges consistently visualizing diverse histological subtypes in the lung, including metastases.

Study limitations include the lack of a control strategy to minimize potential biases in the conduct of the study as well as the small number of patients with cancer-containing lymph nodes, which limited the evaluation of abenacianine for detecting malignant nodes.

Abenacianine, a molecular-targeted imaging agent, features a tumor-targeting moiety that binds to active cysteine cathepsins, enzymes overexpressed in tumor tissue compared with in healthy tissue. The clinical program for abenacianine supports its pan-cancer potential, having identified a wide range of histological subtypes, including NSCLC (adenocarcinoma and squamous cell carcinoma subtypes); large-cell neuroendocrine carcinoma; colorectal, breast, and prostate cancer; carcinoid tumors; sarcoma; and lymphoma. Preclinical studies further support the potential of abenacianine as an IMI agent for additional cancers, including esophageal cancer^8^ and breast cancer.^14^

A key advancement in the design of abenacianine is the incorporation of the NIR fluorescent dye ICG into the structure of abenacianine, ensuring compatibility with all NIR intraoperative imaging systems capable of visualizing ICG. This feature facilitates seamless incorporation into existing surgical workflows. This allows visualization by robotic systems and systems that are available in almost all hospitals in the USA.

Of the patients in this study of IMI with abenacianine, 45% experienced at least one CSE consisting of the localization of a known lung nodule not found intraoperatively using standard techniques, discovery of a synchronous or occult lesion, identification of a close margin, or identification of lymph nodes. Abenacianine appeared safe and well tolerated in this study; no serious drug-related adverse events were observed, and the drug-related adverse events observed in this study were consistent with previous abenacianine phase I and phase II clinical trials.

The results of this study are consistent with those of the previous phase II open-label clinical trial (NCT05400226).^9^ In that study, NIR imaging was used to localize nodules, seek occult lesions, and assess resection margins. Of the 40 patients who underwent pulmonary resection with abenacianine, 17 (42.5%) had at least one CSE.

In many cases, the use of IMI with tumor-targeted NIR imaging agents enhances a surgeon’s ability to localize difficult-to-find lesions, including those not found by standard surgical techniques, achieve adequate margins, and detect cancerous lymph nodes. IMI with abenacianine has the potential to improve surgical outcomes for patients undergoing pulmonary nodule resection. The results presented here support the continued development of abenacianine for use in the surgical resection of cancer in the lung, a phase III study is forthcoming. Clinical work on other indications is also under way.

## Figures and Tables

**Table 1:** Patient characteristics

Characteristic	Overall (*N* = 89)
Age, years	
Mean	66.9
Median	68.0
Minimum; maximum	18; 85
Age, years	
<65	24 (27.0)
≥65	65 (73.0)
Sex	
Male	32 (36.0)
Female	57 (64.0)
Ethnicity	
Hispanic or Latino	9 (10.1)
Not Hispanic or Latino	77 (86.5)
Not reported	1 (1.1)
Unknown	2 (2.2)
Race	
American Indian or Alaska Native	1 (1.1)
Asian	10 (11.2)
Black or African American	4 (4.5)
Caucasian	70 (78.7)
Other	3 (3.4)
Not reported	1 (1.1)
BMI, kg/m^2^	
N	86
Mean	28.08
Median	26.95
Minimum; maximum	18.8; 53.8
Smoking history	
Current	11 (12.4)
Previous	45 (50.6)
Never	33 (37.1)
Missing	0
Primary tumor pathology	
NSCLC—adenocarcinoma	56 (62.9)
NSCLC—squamous cell carcinoma	7 (7.9)
NSCLC—unknown subtype	1 (1.1)
Metastatic disease	21 (23.6)
No malignant tissue—benign lesions only	4 (4.5)

Data are presented as *n* (%) unless otherwise indicated

BMI, body mass index; NSCLC, non-small-cell lung cancer

**Table 2:** Surgical procedures (intent-to-treat population)

Surgical procedure	Number (*N* = 89)
Wedge	43
Segmentectomy	15
Lobectomy	9
Wedge with completion segmentectomy	3
Wedge with completion lobectomy	5
Segmentectomy with completion lobectomy	1
Wedge with completion lobectomy and wedge (different lobe)	1
Segmentectomy and wedge (different segment/lobe)	8
Lobectomy and wedge (different lobe)	4

**Table 3:** Clinically significant events (intent-to-treat population)

Patient-level CSEs (total number of patients = 89)	Patients	% of patients
Patients with at least one CSE	40	44.9
Patients with at least one localization CSE	33	37.1
Patients with at least one synchronous lesion CSE	3	3.4
Patients with at least one margin CSE	8	9.0
Patients with at least one lymph node CSE	1	1.1
Individual CSEs (total number of CSEs = 52)	CSEs	% of CSEs

Localization CSE	37	71.2
Synchronous/occult lesion CSE	3	5.8
Margin CSE	10	19.2
Lymph node CSE	2	3.8

CSE, clinically significant event

**Table 4:** Estimated diagnostic characteristics for cancerous tissue

Characteristic	In vivo imaging (89 patients)	Ex vivo imaging (89 patients)
	Lesions (*n* = 141)	Lesions (*n* = 134)
True positives	90	97
False positives	30	19
True negatives	4	9
False negative	17	9
	Model estimate (95% CI)	Model estimate (95% CI)

Sensitivity	0.841 (0.757–0.900)	0.915 (0.845–0.955)
Positive predictive value	0.750 (0.670–0.816)	0.836 (0.753–0.895)
False discovery rate	0.250 (0.184–0.330)	0.164 (0.105–0.247)

CI, confidence interval

## Data Availability

The data underlying this article cannot be shared publicly because of data protection reasons. The data may be shared upon reasonable request to the corresponding author.
